# Prognostic factors for long-term survival after pancreaticoduodenectomy for periampullary adenocarcinoma. A retrospective cohort study

**DOI:** 10.1016/j.amsu.2020.07.059

**Published:** 2020-08-11

**Authors:** Hazem Zakaria, Ahmed N. sallam, Islam I. Ayoub, Emad H. Gad, Mohammad Taha, Michael R. Roshdy, Dina Sweed, Nahla K. Gaballa, Taha Yassein

**Affiliations:** a Department of Hepatopancreatobiliary and Liver Transplant Surgery, National Liver Institute, Menoufia University, Egypt; b Department of General Surgery, Faculty of Medicine, Minia University, Egypt; c Department of Pathology, National Liver Institute, Menoufia University, Menoufia, Egypt; d Department of Anesthesiology and Intensive Care, National Liver Institute, Menoufia University, Egypt

**Keywords:** Pancreaticoduodenectomy, Periampullary adenocarcinoma, Perineural invasion, Survival, AC, (adenocarcinoma), PAAC, (periampullary adenocarcinoma), PDAC, (Pancreatic duct adenocarcinoma), SD, (standard deviation), PD, (Pancreaticoduodenectomy), DM, (diabetes mellitus), HTN, (hypertension), IHD, (ischemic heart disease), HCV, (hepatitis C virus), HBV, (hepatitis B virus), CA, 19-9(Carbohydrate antigen 19-9), ICU, (intensive care unit), LNs, (lymph nodes), PPPD, (pylorus preserving pancreaticoduodenectomy), PJ, (pancreatico-jejunostomy), PG, (pancreatico-gastrostomy)

## Abstract

**Background:**

Periampullary adenocarcinoma (PAAC) had a poor prognosis, and pancreaticoduodenectomy (PD) remains the only potentially curative treatment. The study aimed to identify the impact of different clinicopathological factors on long-term survival following PD for PAAC.

**Patients and methods:**

This study is a retrospective cohort study for the patients who underwent PD for pathologically proven PAAC from January 2010 to January 2019. Statistical analysis was done using Cox regression multivariate analyses for independent risk factors for survival.

**Result:**

There were 137 patients with PAAC who underwent PD, 79 patients (57.7%) underwent pylorus-preserving PD. Pancreatico-jejunostomy was done in 108 patients (78.8%). The primary analysis showed that risk factors for poor long-term survival include patients with co-morbidities like hypertension or ischemic heart disease, Carbohydrate Antigen 19-9 > 400U/ml, tumor size > 3 cm, poor tumor differentiation, positive lymph nodes invasion, lymphovascular invasion, and Perineural invasion. Multivariate analysis demonstrated that large tumor size > 3 cm (HR: 0.177, 95%CI: 0.084–0.374, P = 0.002), poorly differentiated tumor (HR: 0.059, 95%CI: 0.020–0.0174, P = 0.016), and perineural invasion in the pathological study (HR: 0.101, 95%CI: 0.046–0.224, P = 0.006) were independent risk factors for poor 5-years survival. The prognosis was better in ampullary adenocarcinoma (5-year survival was 42.1%) than pancreatic adenocarcinoma (5-year survival was 24.3%). The 1, 3, 5 and 7-year overall survival rates were 84.5%, 57.4%, 35.9% and 20.1% respectively.

**Conclusion:**

It seems from the current study that Tumor size > 3 cm, poor tumor differentiation, and Perineural invasion were independent predictors of poor survival in patients with PAAC.

## Highlights

• Periampullary adenocarcinoma (PAAC) is highly aggressive tumor.
• It has late diagnosis with poor prognosis.
• Pancreaticoduodenectomy (PD) is still the only curative treatment option.
• PD surgery is associated with high morbidity and mortality.
• It is important to determine which patients who can receive benefit from surgery.

## 1 Introduction

Periampullary adenocarcinoma (PAAC) including adenocarcinoma (AC) of pancreatic head, the distal common bile duct (CBD), the second portion of the duodenum, and the ampulla of Vater, it accounts for approximately 0.2% of all gastrointestinal tract tumors. In recent years, the occurrence of periampullary tumors has an increasing trend although is relatively uncommon neoplasm [[1], [2], [3]].

Pancreaticoduodenectomy (PD) is the treatment of choice for PAAC, however, only 10–15% are resectable at the time of diagnosis. Patient survival after radical resection of periampullary tumors greatly varies, the different biology of the tumor origin could result to some degree into the difference of prognosis [[4], [5], [6]].

Several clinicopathological factors, such as tumor size, resection margin, cell differentiation, lymph node metastasis, perineural and perivascular invasion have been comprehensively studied for determining survival outcome after PD for periampullary cancers [[7], [8], [9]]. Lymphovascular invasion and perineural infiltration in the specimens post-Whipple were reported to be associated with reduced 5-year survival in patients with PAAC [[10], [11], [12]].

Pancreaticoduodenectomy surgery is associated with high morbidity and mortality, therefore it is important to determine which patient can receive benefits from surgery to avoid unnecessary intervention and to facilitate treatment planning of neoadjuvant and adjuvant treatments [12,13]. This study aimed to investigate the prognostic factors for long-term survival in resectable PAAC.

## 2 Patients and methods

We conducted a retrospective study to patients who underwent PD for PAAC between January 2010 to January 2019 at the department of Hepato-pancreato-biliary surgery, National Liver Institute, Menoufia University, Egypt. Data were retrieved from the prospectively collected pancreatic database and patients' medical files, after local Institutional Review Board approval. The research goes with the standards of the Declaration of Helsinki and ethical guidelines and was registered in the clinical trial no ChiCTR2000034782. The study was written in line with the Strengthening the Reporting of Cohort Studies in Surgery (STROCSS) criteria [14]. Patients with confirmed PAAC in the pathological study of the specimen after surgery were included in our study. Other pathological types of lesions after PD were excluded from the study. Data on preoperative, Intraoperative, and postoperative care were collected and analyzed.

### 2.1 Preoperative evaluation

Magnetic resonance image (MRI) or multi-detector abdominal computed tomography (CT) with three-dimensional reconstructions are used to evaluate the periampullary tumors and its relation to vascular structures. Endoscopic ultrasound was done for cases with suspicious diagnosis and for determining the relation of the mass with the surrounding vessels. Preoperative endoscopic retrograde cholangiopancreatography (ERCP) or percutaneous trans-hepatic drainage (PTD) was done in case of cholangitis or delayed surgery.

### 2.2 Surgical procedure and pathological evaluation

**Fig. 1:**
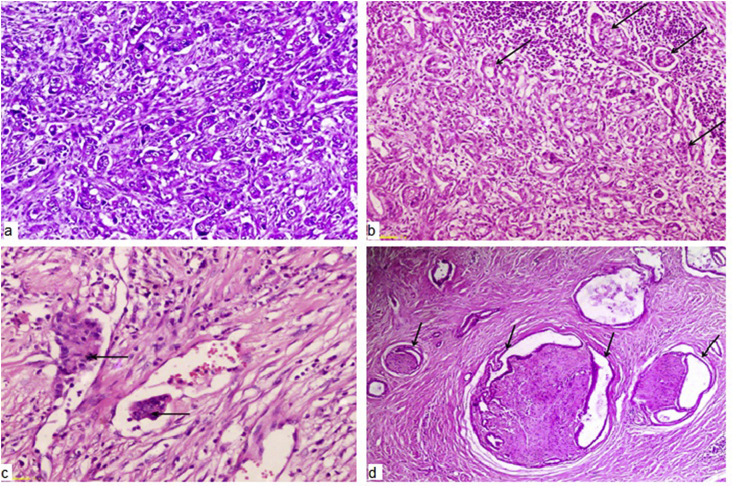
a) Poorly differentiated pancreatic ductal adenocarcinoma (H&E 100x). b) Positive lymph node invasion of Pancreatic ductal adenocarcinoma (black arrows) (H&E 4x). c) Lymph vascular invasion of pancreatic ductal adenocarcinoma (H&E 100x). d) Extensive perineural invasion of well differentiated pancreatic ductal adenocarcinoma (black arrows) (H&E 4x).

Laparotomy was done by bilateral subcostal or midline incision. Patients underwent classical Whipple's operation or pylorus-preserving pancreaticoduodenectomy (PPPD). Pancreatic reconstruction was done by either pancreatico-jejunostomy (PJ) or pancreatico-gastrostomy (PG). Wedge or segmental resection of the portal vein or superior mesenteric vein was performed if a pancreatic head mass was inseparable from the vein. The histopathological features of the specimens were analyzed according to; tumor origin, size, grade, resection margin, lymph node (LN) invasion, perineural and lymphovascular invasion. According to Royal College of Pathologists' guidelines on reporting histological outcomes after major pancreatic resections [15], perineural infiltration was considered positive if tumor cells were identified within the perineural space and/or nerve fibers whereas lymphovascular invasion was defined as the presence of tumor within an endothelial lined or lymphatic space (Fig. 1)**.**

### 2.3 Postoperative follow-up

Follow-up has been arranged in the outpatient clinic or through personal contact, every three months in the first year, in the second and third years every six months then yearly later on. The follow-up was from the date of surgery until July 2020 with a median period of follow up 39 months. Long-term survival was considered ≥ 5-year of survival. Postoperative complications were classified according to the Dindo-Clavien grading system [16]. Postoperative pancreatic fistula (POPF), post pancreatectomy hemorrhage (PPH), and delayed gastric emptying (DGE) were defined and graded according to the International Study Group for Pancreatic Surgery (ISGPS) [[17], [18], [19]].

### 2.4 Statistical analysis

Statistical analysis was done using SPSS 23 (SPSS Inc., Chicago, IL). Fisher's exact or Chi-square X^2^ test was used for categorical variables comparison. For comparing 2 or more continuous variables, the Mann-Whitney *U* test or Kruskal-Wallis test was used respectively. Overall survival (OS) rates in different groups were done by using the Kaplan-Meier method, while the survival rate comparison was applied by the Log-rank test. Cox's regression model was appraised for the multivariate analysis in case of any significant variable in the univariate analysis. *P-*value was considered to be statistically significant if less than 0.05.

## 3 Results

### 3.1 Demographic and preoperative data of the patients

During this study, 137 patients underwent PD for PAAC. Patient demographics and characteristics are shown in Table 1. Of these patients 84 (61.3%) were male and the mean age was 56.8 years. The main complaint was jaundice in 112 patients (81.8%). Preoperative biliary drainage was done in 58 patients (42.3%); by ERCP in 42 patients and PTD in 16 patients.

**Table 1:** Demographic and preoperative data of the patients.

Variables	Patients with PD (n = 137)
**Age (y)**	
mean ± SD	56.8 ± 12.9
(range)	(28–82)
**Sex**	
Male	84 (61.3%)
Female	53 (38.7%)
**Bodyweight**	
mean ± SD	65 ± 17
(range)	(59–105)
**Co-morbidities**	
DM	59 (43.1%)
HTN and/or IHD	42 (30.7%)
Associated HCV or HBV	11 (8%)
Chest problem	13 (9.5%)
**History of smoking**	
Yes	62 (45.3%)
No	75 (54.7%)
**Main symptoms**	
Jaundice	112 (81.8%)
Itching	46 (33.6%)
Loss of weight	62 (45.3%)
Anorexia	64 (46.7%)
Abdominal pain	73 (53.3%)
Vomiting	57 (41.6)
**Preoperative total bilirubin (mg/dl)**	
mean ± SD	12.7 ± 5.4
(range)	(2.1–29)
**Albumin (g/dl)**	
mean ± SD	3.7 ± 0.6
(range)	(3.2–5)
**INR**	
mean ± SD	1.1 ± 0.4
(range)	0.9–1.5
**CA 19-9** (U/mL)	
mean ± SD	512 ± 1247
(range)	(4–5710)
**CEA** (U/mL)	
mean ± SD	6.2 ± 15.3
(range)	1–125
**Preoperative biliary drainage**	
Yes	58 (42.3%)
no	79 (57.7%)

PD (pancreaticoduodenectomy), DM (diabetes mellitus), HTN (hypertension), IHD (ischemic heart disease), HCV (hepatitis C virus), HBV (hepatitis B virus), INR (international normalized ratio), CA19.9 (carbohydrate antigen 19.9), CEA (carcinoembryonic antigen), SD (standard deviation).

### 3.2 Operative, pathological, postoperative data and complications

Seventy-nine patients (57.7%) underwent PPPD and 65.7% patients had PDAC in the pathological study. According to the type of pancreatic reconstruction; PJ was done in 108 patients (78.8%), mainly by duct to mucosa in 68 patients, whereas PG was done in 29 patients (21.2%). Seventeen patients (12.4%) underwent vascular reconstruction; PV or SMV reconstruction was done by lateral venoraphy in 10 patients and end to end primary repair using 6/0 proline in 7 patients. Postoperative pancreatic fistula was found in 26 patients (19%) and mainly grade A POPF. Other operative pathological and postoperative data were shown in Table 2, Table 3.

**Table 2:** Operative and pathological data.

Variables	Patients with PD (n = 137)
**Type of operation**	
PPPD	79 (57.7%)
Classic Whipple	58 (42.3%)
Pancreatic texture	
Firm	57 (41.6%)
Soft	80 (58.4%)
**Type of pancreatic reconstruction**	
Pancreaticogastrostomy	29 (21.2%)
Pancreaticojejunostomy	108 (78.8%)
- invagination	40 (37%)
- duct to mucosa	68 (63%)
**Pancreatic duct stent**	
Yes	46 (33.6%)
No	91 (66.4%)
**Vascular reconstruction**	
Yes	17 (12.4%)
No	120 (87.6%)
**Operative time** (min)	
mean ± SD	450 ± 70
(range)	(280–560)
Operative blood loss	
mean ± SD	900 ± 550
(range)	(300–2200)
**Blood transfusion** (unit)	
mean ± SD	1.5 ± 1
(range)	(0–5)
**Site of the tumor**	
Pancreatic head	90 (65.7%)
Ampullary	31 (22.6%)
Lower CBD	10 (7.3%)
Duodenum	6 (4.4%)
**Pathological maximum tumor diameter**	
Mean ± SD	3.4 ± 1.6
(Range)	(1.4–9)
**Tumor stage**	
T1	12 (8.8%)
T2	57 (41.6%)
T3	52 (38%)
T4	16 (11.6%)
**Tumor differentiation**	
Well/moderate	103 (75.2%)
Poor	34 (24.8%)
**Positive lymph node**	
Yes	58 (42.3%)
No	79 (57.7%)
**Number of LN dissection mean (range)**	**5(2-24)**
**Number of LN infiltration mean (range)**	0(0-5)
LN ratio	
0	79 (57.7%)
<0.2	18 (13.1%)
0.2–0.4	26 (19%)
>0.4	14 (10.2%)
**lymph vascular invasion**	
Yes	62 (45.3%)
No	75 (54.7%)
**Perineural invasion**	
Yes	51 (37.2%)
No	86 (62.8%)
**Positive surgical margin**	
Yes	13 (9.5%)
No	124 (90.5%)

PD (pancreaticoduodenectomy), PPPD (pylorus preserving pancreaticoduodenectomy), CBD (common bile duct), SD (standard deviation). LN (lymph nodes).

**Table 3:** Postoperative data and complications.

Variables	Patients with PD (n = 137)
Post-operative complications	
- Postoperative pancreatic leak - grade A - grade B - grade C	26 (19%) 13 8 5
- Post-pancreatectomy hemorrhage	9 (6.6%)
- Biliary leak	10 (7.3%)
- Delayed Gastric Empty	20 (14.6%)
- Wound infection	26 (19%)
- Pulmonary complications	12 (8.8%)
**Reoperation**	
Yes	14 (10.2%)
No	123 (89.8%)
**ICU stay** (days)	
mean ± SD	3 ± 2
(range)	(1–9)
**Hospital stay** (days)	
mean ± SD	13 ± 3
(range)	(10–19)
**Hospital mortality**	9 (6.6%)
**Postoperative chemo and/or radiotherapy**	
Yes	81 (59.1%)
No	56 (40.9%)
**Recurrence of tumor**	34/128 (26.6%)
Clavien Dindo grades of complications	
0	40 (29.2%)
I	30 (21.9%)
II	28 (20.4%)
IIIa	13 (9.5%)
IIIb	11 (8.1%)
IVa	4 (2.9%)
IVb	2 (1.5%)
V	9 (6.5%)

PD (pancreaticoduodenectomy), ICU (intensive care unit), SD (standard deviation).

### 3.3 Risk factors for survival

**Fig. 2:**
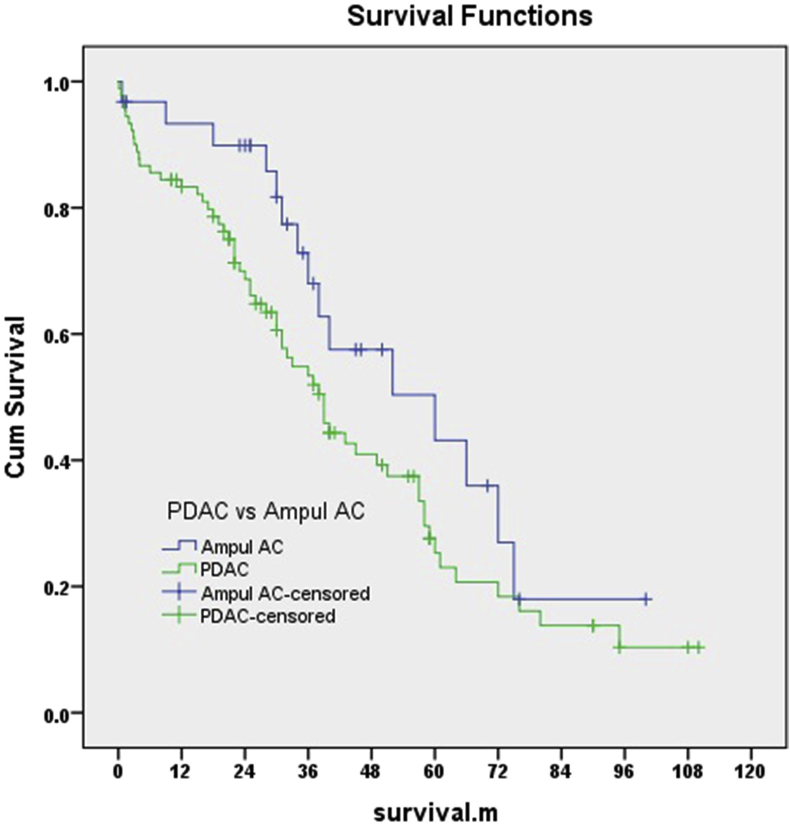
Kaplan Meier curves for overall survival in patients with PDAC and ampullary adenocarcinoma.

The median survival across all patients was 33 months; 26 months for patients with PDAC (5-year survival was 24.3%), 37 months for ampullary adenocarcinoma (5-year survival was 42.1%) (Fig. 2). The 1-, 3-, 5- and 7-year tumor-free survival was 80.1%, 49.3% 31.6%, and 18.6%respectively, while the 1-, 3-, 5- and 7-year overall survival was 84.5%, 57.4%, 35.9% and 20.1% respectively.

In univariate analysis (Table 4) the potential risk factors for poor survival were, preoperative comorbidity like hypertension (HTN) or ischemic heart disease (IHD) (P = 0.02), high preoperative carbohydrate antigen 19-9 (CA19-9) (P = 0.04), tumor diameter > 3 cm (P = 0.001), poor tumor differentiation (P = 0.001), LN invasion (P = 0.04), lymphovascular invasion (P = 0.05), and perineural invasion (P = 0.001). In multivariate analysis (Table 5) the independent risk factors for poor survival were, large tumor size > 3 cm (HR: 0.177, 95%CI: 0.084–0.374, P = 0.002), poorly differentiated tumor (HR: 0.059, 95%CI: 0.020–0.0174, P = 0.016), and presence of perineural invasion in the pathological study (HR: 0.101, 95%CI: 0.046–0.224, P = 0.006).

**Table 4:** Univariate analysis for potential risk factors for survival.

Variables	Number of Deaths in PD per cases observed	% of Deaths	P-value
**Age**			0.11
>60	31/51	60.8%	
≤60	40/86	46.5%	
**Gender**			0.71
Male	46/84	54.8%	
Female	25/53	47.2%	
**Co-morbidities**			
-DM			
Yes	28/59	47.5%	0.57
No	43/78	55.1%	
-HTN/IHD			
Yes	28/42	66.7%	**0.02**
No	43/95	45.3%	
**Total bilirubin**			0.39
**>**10 mg/dl	31/53	58.5%	
≤10 mg/dl	40/84	47.6%	
**Preoperative biliary drainage**			0.95
Yes	30/57	52.6%	
No	41/80	51.3%	
**CA 19-9** (U/ml)			**0.04**
>400	27/41	65.9%	
≤400	44/96	45.8%	
**Pancreatic texture**			0.09
soft	32/70	45.7%	
Firm	39/67	58.2%	
**Type of pancreatic reconstruction**			0.26
Pancreatico-gastrostomy	12/29	41.4%	
Pancreatico-jejunostomy	59/108	54.6%	
**Vascular reconstruction**			0.07
Yes	12/17	70.6%	
No	59/120	49.2%	
**Operative time (min)**			0.48
>420	43/79	54.4%	
≤420	28/58	48.3%	
**Operative blood loss (ml)**			0.17
>1000	33/56	58.9%	
≤1000	38/81	46.9%	
**Blood transfusion**			0.09
Yes	30/49	61.2%	
no	41/88	46.6%	
**Postoperative pancreatic fistula**			0.68
yes	10/21	47.6%	
No	61/116	52.6%	
**Maximum tumor diameter (cm)**			**0.001**
>3	41/57	71.9%	
≤3	30/80	37.5%	
**Tumor origin**			0.79
Pancreatic head	44/90	48.9%	
Ampullary	18/31	58.1%	
Lower CBD	6/10	60%	
Duodenum	3/6	50%	
**Tumor differentiation**			**0.001**
Good/moderate	43/103	41.7%	
Poor	28/34	82.4%	
**Resection margin**			0.67
R0	65/124	52.4%	
R1 or R2	6/13	46.2%	
**Positive lymph nodes**			**0.04**
Yes	36/58	62.1%	
No	35/79	44.3%	
**lymph vascular invasion**			**0.05**
Yes	38/62	61.3%	
No	33/75	44%	
**Perineural invasion**			**0.001**
Yes	36/51	70.6%	
No	35/86	40.7%	

PD (pancreaticoduodenectomy), DM (diabetes mellitus), HTN (hypertension), IHD (ischemic heart disease), CA19.9 (carbohydrate antigen 19.9), CBD (common bile duct), SD (standard deviation).

**Table 5:** Multivariate analysis of independent risk factors for survival in PD.

Variable	HR	95%CI		*P*-value
**HTN/IHD**	0.579	0.269	1.246	0.162
**CA19**–**9** > **400 u/ml**	0.950	0.588	1.535	0.833
**Tumor size** > **3 cm**	0.177	0.084	0.374	**0.001**
**Poorly differentiated tumor**	0.059	0.020	0.174	**0.016**
**Lymph nodes invasion**	0.677	0.187	2.458	0.553
**Lymph vascular invasion**	2.462	0.232	26.144	0.455
**Perineural invasion**	0.101	0.046	0.224	**0.006**

PD (pancreaticoduodenectomy), HTN (hypertension), IHD (ischemic heart disease), CA19.9 (carbohydrate antigen 19.9).

## 4 Discussion

Pancreaticoduodenectomy operation remains the standard curative approach for periampullary tumors. Despite several refinements in the surgical technique with the improvement of postoperative mortality, the long-term prognosis still disappointing with a 5-years survival rate rarely to exceed 20% in some centers. These results raised the enthusiasm to search for the main factors that can improve the prognosis of periampullary tumors with the optimal resection [1,4,6,12].

In the PAAC the long-term survival rate varies in a wide range related to the different anatomical locations in the periampullary region. El Nakeeb et al., in their study, showed that 5-year survival was 20.6% in PAAC with a median survival of 34 months. The worst prognosis was reported in pancreatic head AC with 5%–20% 5-year survival, and a better prognosis was in ampullary and duodenal AC with 5-year survival 30%–65% [20]. Also, Zakaria et al., demonstrated that 5-year survival rate in patients with PDAC was 23.4% [21].

Other studies have reported that there is a comparatively favorable prognosis among PAAC, with 5-year OS rates of 30–70% after radical resection and adjuvant chemo-radiation therapy [[22], [23], [24]]. Feretis et al., reported in their study that the overall 1-, 3-, and 5-year survival rates were 79.8%, 42.2%, and 34.9%, respectively [25], while He et al., demonstrated in their study that 1-, 3-, and 5-year OS rates were 88.2%, 66%, and 53%, respectively [26]. Our study goes parallel with these previous studies with comparable results.

Serum CA19-9 has manifested as a clinically valuable biomarker of pancreatic cancer, and it has proved that higher serum CA19-9 level preoperatively can predict poorer survival of pancreatic cancer after resection [27]. However, there were few studies on the prognostic value of CA19-9 in periampullary cancer. Gao et al. have suggested that periampullary cancer patients with preoperative serum CA19-9 > 35 U/ml are prone to have a poorer survival [28]. Also, El Nakeeb et al., showed that preoperative serum CA19-9 > 37 U/ml was associated with a poor survival rate [20]. In the present study the elevated CA 19-9 (>400U/ml) had a statistical significance risk for poor survival in univariate analysis, similar to the previous studies.

According to patients with IHD, a previous investigation reported that there was an association between IHD and mortality after PD that did not remain significant in the multivariate model [29], as seen in our study.

Tumor size is a well-established predictor of survival. In general tumor size <3 cm has a better prognosis. In some studies it was only significant in univariate analysis [30]. Other studies, reported that the size of the tumor was independent predictors of survival [22]. In our study, both univariate and multivariate analysis demonstrated that tumor size > 3 cm was significantly independent risk factor for poor survival.

Venous reconstruction can be done if there is an invasion of porto-mesentric access to achieve R0 resection with accepted postoperative morbidity and mortality. Some studies showed that the resection margin was an independent risk factor for survival, and R0 achieved significantly better OS [6,7,20,31]. In contrast, the meta-analysis study by Butturini et al., found that resection margin was not a significant prognostic factor for survival [32] as seen in our study, it may be due to the difference in the pathological definitions and findings of the resection margin.

Also, tumor differentiation has been reported to be associated with the progression of PAAC. Most studies in the multivariate analysis reported that poor tumor differentiation was a poor prognostic factor for survival [26,33,34], similarly, to what is seen in our series.

Other histopathological characters like lymph node metastasis and lymphovascular invasion should be regarded as an independent predictor of survival and may have therapeutic and prognostic implications for patients [11,33,34]. The poor OS reported in the study by Al-Jumayli et al., was likely due to the high rate of tumor invasion and extension [35]. As the tumor grows along nerves in the pancreas, it infiltrates distally to follow an arterial channel, reducing the chances of complete microscopic clearance [36]. Zhao et al., reported in their study that the perineural infiltration was a significant prognostic factor after pancreatic head resection and has been proven to be related to local failure [37]. The perineural invasion appeared to be the most significantly associated with 1-year mortality [10,12,38]. In our study, the perineural invasion was significantly independent risk factors for poor OS.

Panaro et al., concluded that PDAC is considered a systemic disease, and microvascular invasion is a major prognostic factor after PD as it can lead to distant metastasis, but unfortunately, we cannot predict microvascular invasion in the preoperative image, so it raised the question about the significance of neoadjuvant therapy for all resectable pancreatic cancer, that needs further studies [12].

The limitations of this study are its retrospective nature and single-center experience that is liable for statistical bias, PD surgery was done by different surgeons but they almost have equal experience, the biological behavior of the different PAAC that may have also racial variations may affect the result between centers and needs further study and there is no complete data about the postoperative adjuvant therapy.

**In conclusion**: It seems from the current study that the predictors of poor long-term survival in patients with PAAC were patients with co-morbidities like HTN or IHD, CA19-9 > 400U/ml, tumor size > 3 cm, poor tumor differentiation, LNs invasion, lymphovascular invasion, and Perineural invasion. However, after multivariate analysis tumor size > 3 cm, poor tumor differentiation, and Perineural invasion were independent risk factors of poor long-term survival. Patients with ampullary AC had better mean survival than patients with pancreatic AC.

## Provenance and peer review

Not commissioned, externally peer reviewed.

## Statement of ethics

The research was conducted ethically in accordance with the World Medical Association Declaration of Helsinki. The patients have given their written informed consent on admission and pre-operatively to use their prospective database and files for research work. The study protocol was approved by the National Liver Institute committee and review board.

## Consent

The work has been approved by the National Liver Institute ethical committees, in which the study was performed and the patients gave informed consent to use their retrospectively collected data from files for study and research work.

## Financial support

No.

## Sources of funding

No funding

## Author contribution

Hazem Zakaria 1, Ahmed N sallam1, Islam I Ayoub1, Emad H Gad 1, Mohammad Taha 1, Michael R Roshdy2, Dina Sweed3, Nahla K Gaballa 4, Taha Yassein1.

Had actively participated in the preparation, study design, collection of the data and editing of the manuscript. Statistical analysis was done by Hazem Zakaria.

## Research registration number

Name of the registry: Chinese Clinical Trial Registry.

Unique Identifying number or registration ID: ChiCTR2000034785.

Hyperlink to the registration (must be publicly accessible):

http://www.chictr.org.cn/edit.aspx?pid=55259&htm=4

http://www.chictr.org.cn/listbycreater.aspx

## Guarantor

***Hazem Mohamed Zakaria,*** Department of Hepatopancreatobiliary & liver transplant surgery, National Liver Institute, Menoufia University, 32511 Shebin El-koom, Menoufia, Egypt.

**E-mail:** hazemlasheenn@yahoo.com.

Tel: +2 01019353448.

**Fax:** +20482234586; Tel.: +20482222740.

## Declaration of competing interest

No conflict of interest.
